# A State-of-the-Art Review on Recent Biomedical Application of Polysaccharide-Based Niosomes as Drug Delivery Systems

**DOI:** 10.3390/polym17111566

**Published:** 2025-06-04

**Authors:** Andreea-Teodora Iacob, Andra Ababei-Bobu, Oana-Maria Chirliu, Florentina Geanina Lupascu, Ioana-Mirela Vasincu, Maria Apotrosoaei, Bianca-Stefania Profire, Georgiana-Roxana Tauser, Dan Lupascu, Lenuta Profire

**Affiliations:** 1 Department of Pharmaceutical and Therapeutic Chemistry, Faculty of Pharmacy, University of Medicine and Pharmacy “Grigore T. Popa” of Iasi, 16 University Street, 700115 Iasi, Romania; andreea.panzariu@umfiasi.ro (A.-T.I.); oana-maria.ionescu@umfiasi.ro (O.-M.C.); florentina-geanina.lupascu@umfiasi.ro (F.G.L.); ioana-mirela.vasincu@umfiasi.ro (I.-M.V.); apotrosoaei.maria@umfiasi.ro (M.A.); roxana.tauser@umfiasi.ro (G.-R.T.); dan.lupascu@umfiasi.ro (D.L.); lenuta.profire@umfiasi.ro (L.P.); 2 Department of Internal Medicine, Faculty of Medicine, University of Medicine and Pharmacy “Grigore T. Popa” of Iasi, 16 University Street, 700115 Iasi, Romania

**Keywords:** niosomes (NIOs), drug delivery systems, polysaccharide, medical and pharmaceutical applications

## Abstract

The development of nanocarriers for drug delivery has drawn a lot of attention due to the possibility for tailored delivery to the ill region while preserving the neighboring healthy tissue. In medicine, delivering drugs safely and effectively has never been easy; therefore, the creation of surfactant-based vesicles (niosomes) to enhance medication delivery has gained attention in the past years. Niosomes (NIOs) are versatile drug delivery systems that facilitate applications varying from transdermal transport to targeted brain delivery. These self-assembling vesicular nano-carriers are formed by hydrating cholesterol, non-ionic surfactants, and other amphiphilic substances. The focus of the review is to report on the latest NIO-type formulations which also include biopolymers from the polysaccharide class, highlighting their role in the development of these drug delivery systems (DDSs). The NIO and polysaccharide types, together with the recent pharmaceutical applications such as ocular, oral, nose-to brain, pulmonary, cardiac, and transdermal drug delivery, are all thoroughly summarized in this review, which offers a comprehensive compendium of polysaccharide-based niosomal research to date. Lastly, this delivery system’s limits and prospects are also examined.

## 1. Introduction

L’Oreal was the pioneering firm to document the presence of non-ionic surfactant vesicles as a distinctive feature of the cosmetics sector throughout the 1970s and 1980s. Lancôme, a subsidiary of L’Oreal, introduced its inaugural product, “Niosome”, in 1987 [1,2]. 

**Figure 1:**
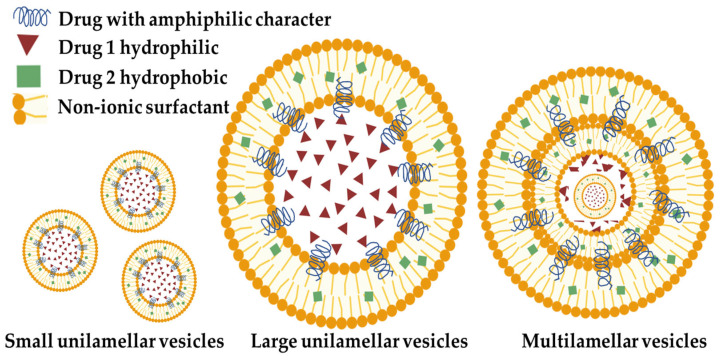
Schematic representation of NIO (made with the help of BioRender.com).

At present, niosomes (NIOs) are being investigated as innovative and effective drug carriers, providing various benefits in comparison to traditional liposomes, including improved chemical stability, reduced cost, simplified manufacturing, and increased formulation flexibility [3,4]. Niosomal systems provide two main modalities for targeted medication delivery: (1) passive targeting of the reticuloendothelial system (RES) which involves phagocytic cells in reticular connective tissue and (2) coupling with functionalized ligands for targeted delivery to specific organs and tissues [5]. Notably, NIOs have the ability to encapsulate both hydrophilic and lipophilic active substances but also substances that have an amphiphilic character (Figure 1) within their inner nucleus and outer bilayers. Hence, they can serve as vehicles for the transportation of various pharmaceuticals, hormones, and genetic material such as antigens [6]. 

NIOs, as a nanovesicular drug carrier [7,8], offer several benefits in transdermal drug administration. These advantages include prolonged drug release, improved penetration, increased skin retention, lower preparation costs, and enhanced stability. Consequently, NIOs have potential applications in the beauty industry [9]. NIOs are utilized in cosmetics and skincare products through the enhancement of skin penetration of chemicals. This is achieved by their ability to reversibly decrease the barrier resistance of the horny layer, thereby enabling the component to enter the living tissues at a higher rate [10,11].

NIOs, owing to their lipophilicity and nanoscale vesicular structure, can interact with the nasal mucosa, hence facilitating increased penetration. Niosomal preparations have superior brain targeting effectiveness compared to orally marketed tablets. It was shown that intranasal (*i.n.*) NIOs yield about a 6.47-fold enhancement in cerebral availability compared to the same formulation delivered orally, so indicating a substantial role of the direct nose-to-brain route in brain targeting [12]. Regarding the ocular administration, NIOs offer several advantages over other ocular drug delivery systems (DDSs), including: (i) facile and economical fabrication; (ii) chemical stability; (iii) low toxicity and high compatibility due to their non-ionic characteristics; (iv) improved solubility and permeability facilitated by the use of surfactants. Therefore, NIOs are formulated to enhance the bioavailability of the active substance, and it has been reported that drugs can permeate the cornea more effectively after being incorporated into NIOs, especially when they are coated with a polysaccharide, e.g., hyaluronic acid [13,14].

Recent studies demonstrated that the *i.v.* administration of niosomal formulation resulted in a significant reduction in cardiac biomarkers and lipid peroxidation levels, indicating their capacity to alleviate the negative impacts of cardiotoxicity, thus making them a promising cardiac drug delivery system [15]. In regard to the use of NIOs as efficient non-viral vectors for augmented gene transfer to human mesenchymal stem cells (MSCs), it was reported that they are suitable for gene delivery applications, offering new approaches for the prevention and/or treatment of various diseases [16]. As for the development of niosomal DDSs used in cancer therapy, these formulations provide significant promise to transform contemporary cancer therapies by facilitating precise and focused drug release at cancer sites, hence reducing the adverse effects linked to conventional chemotherapy [17].

Polysaccharides are an essential source of versatile materials regarded as superior to other polymers because of their advantageous features, including homogeneity, bio-adhesion, and bioactivity [18,19]. Traditional drug delivery systems are characterized by inadequate selective distribution, limited solubility, quick clearance, and harm to surrounding healthy tissues. Consequently, DDSs are extensively employed to enhance bioavailability and mitigate therapeutic side effects by targeting the administration of pharmaceuticals to specific sites. In recent years, polysaccharides have been extensively utilized in medication delivery due to their nontoxicity, stability, accessibility, practicality, and cost-effectiveness [20]. DDSs derived from polysaccharides represent a category of biocompatible DDSs characterized by polysaccharide macromolecules as the structural backbone. These polysaccharide-based DDSs, due to their complex formulation, are capable of sensitively detecting alterations in reactive oxygen species (ROS), pH, and glutathione levels in their surroundings, while also responding to stimuli such as heat, light, and magnetism. Consequently, they can specifically react to the pathological microenvironment to facilitate the release of the encapsulated drug. Responsive DDSs utilizing polysaccharides possess both the pharmacological efficacy of polysaccharides and the benefit of precisely focused drug administration [20,21]. Biopolymer/polysaccharide-based niosomal formulations have several advantages: non-toxicity, ease of processing, exceptional biocompatibility, a high degree of biodegradability, and antibacterial properties, as exemplified by chitosan [22].

This review centers on enumerating the recent pharmaceutical and medical applications of vesicular release systems in the form of NIOs based on different polysaccharides, emphasizing the beneficial role of polysaccharides in the release mode of active substances incorporated in the niosomal structure. The novelty of this work is the focus placed on the use of the biopolymers from the polysaccharide class in order to formulate NIOs as various drug delivery systems (DDSs) with conventional, controlled, or targeted release.

## 2. Non-Ionic Surfactant Vesicles (Niosomes)—Definition and Main Characteristics

NIOs are nanoscale drug delivery systems, similar to liposomes, which distinguish themselves by their bilayer membrane consisting of non-ionic surfactants (in addition to cholesterol) in lieu of phospholipids [23].

The primary constituent of NIO particles is non-ionic surfactants, which possess hydrophilic non-charged heads and hydrophobic tails [24]. The surfactants that can be used in the formulation of NIOs are vast varying from alkyl ethers such as the Brij family or alkyl esters such as the Span and Tween family or esters such as sorbitan ester and crown ester [25,26]. NIOs are generated by the self-assembling of non-ionic surfactants and/or charge enhancers that encapsulate drugs within vesicles [27]. These vesicles consist of colloidal particles that are formed when non-ionic surfactants and pharmaceutical components spontaneously form a tiny lamellar bilayer structure in aqueous fluids [28]. These vesicular delivery systems range in size from 10 nm to 2 μm in diameter. Small unilamellar vesicles have a size ranging from 25–50 nm, multilamellar vesicles range from 50 nm, and large unilamellar vesicles range from 100 nm ([10], Figure 1). Within this bilayered structure is a central empty space [29].

## 3. Classification of the Non-Ionic Surfactant Vesicles (NIOs)

NIOs can be classified according to several criteria, namely: their size, their composition, their appearance, or certain specific characteristics. A variety of NIOs have been documented in the literature. The main types of NIO cited in the literature and their main properties or biomedical applications will be described below (Table 1) [26,30,31].

(a) ***Polyhedral niosomes (polyhedral NIOs)***

An interesting category of NIOs is polyhedral NIOs, characterized by their non-uniform structure and composed of circular vesicles. Polyhedral nanosomes are characterized by having 4–12 aligned sides of equal length. The modification of NIOs using polyethylene glycol is employed to enhance their absorption from the mononuclear phagocytic immune system [41]. Their characteristics have been documented since the end of the 1990s. Polyhedral NIOs exhibit a reversible morphological change into spherical forms when heated beyond their phase conversion temperature. The viscosity of polyhedral NIOs at ambient temperature is greater than that of their spherical equivalents because of their faceted and slightly inflexible structure, and is more influenced by temperature, owing to the process of shape change. The gel phase membranes of polyhedral NIOs at room temperature exhibit greater rigidity and reduced osmotic sensitivity. Nevertheless, their membranes are more permeable due to the absence or low concentration of cholesterol [42,43]). Polyhedral vesicles have demonstrated stability for a minimum of 36 days and possess the capability to entrap and gradually discharge water-soluble markers, including nucleotides and carboxyfluorescein. Luteinizing hormone-releasing hormone (LHRH)-loaded spherical and polyhedral NIOs were developed as slow-release depot systems for intramuscular administration. Spherical conventional NIOs exhibited greater membrane stability compared to polyhedral NIOs, indicating that conventional NIOs serve as more efficient intramuscular depots [32].

(b) ***Proniosomes (proNIOs)***

Experimental research has demonstrated that proNIOs serve as a dependable promoter for the synthesis of NIOs, which can then be used in diverse pharmaceutical delivery systems [2]. ProNIOs can be defined as liquid crystalline forms of NIOs located in a dry condition. Reconstitution of the proNIOs with water leads to a reorganization of the surfactants and other constituents in the formulation, transforming them into NIOs. ProNIOs can exist in either a desiccated powder or gel state, contingent upon the manner of manufacture [44]. NIOs produced from proNIOs are considered superior alternatives to conventional vesicular delivery methods because of their favorable physicochemical characteristics and enhanced chemical durability. ProNIOs provide enhanced versatility in terms of transportation, storage, distribution, and dosage, thereby establishing dry NIOs as a highly adaptable commercial product [45]. Successful nanoencapsulation of resveratrol as a proNIO has been described, where the particle size and form of the proNIOs are determined by the choice of wall material. The proNIOs derived from maltodextrin exhibited a smooth surface, a spherical form, and clusters of individual nanoparticles. In contrast, the proNIOs derived from lactose and pullulan were flaky with rough edges and clustered [46]. Their capacity to effectively address the physical instability challenges might enhance their flexibility in processing, distribution, storage, and dosing [47].

In a 2017 study, proNIOs of risperidone were prepared, optimized, and investigated for their potency in transdermal delivery to address the bioavailability challenges associated with orally administered risperidone. ProNIOs were formulated utilizing different sorbitan esters in combination with cholesterol and soya lecithin, followed by assessment of in vitro characteristics, ex vivo permeation, and in vivo performance. The results demonstrated that the vesicles exhibited a spherical morphology, with sizes varying from 284.00–941.40 nm, and displayed a high zeta potential. The values obtained for the entrapment efficiency for spans exceeded that of tweens. The in vitro release study demonstrated that formulations containing spans display a controllable release profile consistent with the Higuchi model. The relative bioavailability following transdermal administration of proNIOs was 92%, with a tmax of 8 h. The developed proNIO formulation is concluded to be a promising option for enhancing the bioavailability of risperidone [48].

(c) ***Elastic niosomes (elastic NIOs)***

Manosroi et al. describes the formulation of elastic NIOs loaded with papain using the thin-film hydration method with sonication, where Tween 61, cholesterol, and sodium cholate were dissolved in chloroform/methanol, evaporated, and hydrated with papain solution. The main role in elastic NIOs formation is attributed to sodium cholate, since without the addition of cholate non-elastic NIOs have been reported. Following 28 days of application, the gel embodying papain encapsulated in elastic NIOs demonstrated a greater reduction in hypertrophic scars on the induced scars of rabbits’ ears, as measured by a vernier caliper, compared to the gel containing free papain and the gel embodying papain encapsulated in non-elastic NIOs. This study demonstrates that elastic NIOs provide a superior transdermal delivery system for papain, enhancing skin permeation and reducing hypertrophic scars, indicating potential for development as an effective topical scar pharmaceutical product [33].

(d) ***Transfersomes***

Transfersomes represent the most advanced dermal and transdermal carriers currently available. They are liposome-like vesicles characterized by a hydrophilic core encased in a hydrophobic bilayer. The inclusion of an additional surfactant within the phospholipid structure enhances flexibility [49]. Transfersomes provide various advantageous characteristics for direct surface application, including elasticity and flexibility. These characteristics are influenced by the type of edge activator and also by the phosphatidylcholine components. They have demonstrated the potential to release therapeutic substances in a regulated manner, improve the penetration of the skin, and have become a promising option for transdermal drug administration because of their exceptional deformability when traversing the skin [50]. A study compared melatonin encapsulated in transfersomes with free melatonin solution regarding characteristics such as appearance, zeta potential, size, polydispersity index (PDI), percent entrapment efficiency (EE%), and melatonin release and permeation. The incorporation of melatonin into transfersomes demonstrated notable enhancements in physical properties. Specifically, the vesicle size of melatonin-loaded transfersomes was reduced to approximately 120 nm, and the entrapment efficiency achieved 75%. The study concluded that melatonin-incorporated transfersomes exhibit notable anti-inflammatory and collagen synthesis action in vitro. The results indicate that the developed transfersomes may have applications in the pharmaceutical and cosmeceutical fields [51].

(e) ***Bilosomes***

***Bilosomes*** are closed bilayer nanovesicles that contain non-ionic surfactants and bile salts, distinct from transfersomes which improve penetration by breaking down lipid double layers using edge activators [52]. Bilosomes, due to their distinctive composition including bile salts, exhibit particular processing characteristics and physical durability. In the last 10 years, comprehensive studies have emphasized bilosomes’ potential as superior vesicular carriers compared to liposomes and NIOs. Progress in this domain has resulted in the creation of modified bilosomes, including probilosomes and surface-modified bilosomes, thereby augmenting their functionalities and therapeutic efficacy [53]. The integration of bile salts into bilosomes results in the formation of bile bodies, which effectively shield liposomal vesicles from bile acids and prolong their duration at the distribution site. Bile bodies function as a regulated slow-release mechanism, facilitating targeted delivery in the small intestine and mitigating the issue of rapid and excessive substance release. This targeted release improves absorption and guarantees optimal therapeutic efficacy. The formulation of bilosomes to enhance the transdermal delivery of valsartan was reported. The thin-film hydration method, combined with ultrasonication, significantly improved the quality of valsartan bilosome formulations [36].

Sultan et al. developed doxorubicin (DOX) encapsulated in bilosomes modified by the incorporation of efflux inhibitors such as dipyridamole and piperine. The capacity of bilosomes to improve intestinal absorption and medication intake by tumor cells is contingent upon the ability of their constituents to fluidify cellular membranes and escape efflux transporters. The bilosomal co-incorporation of DOX and enhancers showed also an improved cytotoxic activity [54].

A recent study reported the development of an optimal fluticasone propionate-loaded bilosome via the Draper–Lin small composite design, focusing on the optimization of four elements: (1) entrapment efficiency, (2) vesicle size, (3) skin flux, and (4) skin accumulation. Bilosomes were characterized in relation to the drug suspension, incorporated into a carbopol gel, and a histopathological evaluation was performed on carrageenan-induced rat joint arthritis, compared to a traditional gel. Following 20 days of daily application of bilosomal gel for tibio-femoral joint arthritis, the histological construction of the joint and the values of TNF-α and IL-1β normalized. Fluticasone propionate-loaded bilosomes present a promising therapeutic approach for various inflammatory diseases, particularly arthritis, through the transdermal route [55].

Another study on bilosome formulation also aimed at improving transdermal delivery, but this time of niflumic acid (NA), a nonsteroidal anti-inflammatory drug (NSAID), together with Brij. Brij bilosome with niflumic acid (Brij-BNA) gel demonstrated a sustained in vitro release profile and exhibited enhanced ex vivo skin permeation compared to niflumic acid gel. The in vivo study of Brij-BNA gel demonstrated regenerative potential and chondroprotection against monosodium iodoacetate-induced osteoarthritis, with normal histological findings in comparison to NA gel [56]. Other recent bilosome formulations are depicted in Table 2.

(f) ***Discomes***

The formulation of discomes by the incubation of preformed spherical NIOs in Solulan C24 (cholesteryl poly-24-oxyethylene ether) at 75 °C for one hour in order to create discomes with a small cholesterol concentration < 30% mol/mol was reported. As a result, enormous, multidimensional vesicular systems (11–60 mm) were formed. Discomes as an ocular delivery mechanism for timolol maleate have only been reported once. When compared to a timolol maleate solution, the produced discomes have been shown to enhance the ocular bioavailability and entrap a comparatively significant amount of timolol [64]. The requirement for a comparatively high temperature during discome synthesis, which may impact the chemical steadiness of some thermo-labile medicinal drugs, is one potential explanation for this shortage. However, because of the cholesterol, it has been demonstrated that spherical NIOs have more stable membranes than polyhedral ones [37]. By stopping the vesicles at the discosome phase, discosomes—giant, disc-shaped structures—were created from NIOs. Compared to conventional ocular drug delivery methods, they provide all the advantages that come with their unique size [65].

(g) ***Aspasomes***

Aspasomes are characterized as vesicles composed of ascorbyl palmitate (AP), cholesterol, dicethyl phosphate (DCP), and a charge inducer. AP, an ester of ascorbic acid, commonly referred to as vitamin C, is a potent antioxidant and free radical scavenger that enhances cellular health. In contrast to water-soluble ascorbic acid, it possesses superior skin penetration, it may be retained in the lipid cell membrane until the body is prepared, and it exhibits enhanced stability owing to its amphiphilic nature. The suppleness of the skin can be enhanced by stimulating collagen formation through the presence of AP in the composition of aspasomes [66,67]. In 2019, Aboul-Einien et al. published an article about the development and assessment of modified aspasomes to enhance the transdermal delivery of hydrophilic drugs. The modified aspasomes utilized lecithin derived from natural sources instead of dicetyl phosphate. These aspasomes exhibited enhanced loading efficiency and drug retention in the deeper layers of the epidermis [68,69]. 

The encapsulation of melatonin in aspasomes optimized its therapeutic efficacy in androgenic alopecia, as shown by clinical assessment criteria. The favorable properties of aspasomes in the treatment of androgenic alopecia can be attributed to their capacity to enhance melatonin distribution to hair follicles, as well as their interaction with skin lipids and the development of depots inside the skin [70].

In a recent study, an aspasomal cream containing itraconazole (ITZ) was developed, demonstrating enhanced nanocarrier–skin availability, as well as better skin retention and deposition. Furthermore, significant in vivo skin tumor suppression, along with an excellent antioxidant profile, was also facilitated by aspasomal cream. The findings indicated the remarkable efficacy of the specified ITZ aspasomes and associated cream in combating skin cancer, positioning ITZ as either an independent treatment or as a complementary therapy in conjunction with other pertinent medications [71].

(h) ***Bola niosomes (bola NIOs)***

Bola NIOs are synthesized with a specific surfactant known as α,ω-hexadecyl-bis-(1-aza-18-crown-6). Bola surfactant is a non-ionic surfactant initially discovered in the membranes of archaebacteria and possesses the ability to assemble in the presence of cholesterol. These colloidal vesicles are potential vehicles for topical drug administration, exhibiting appropriate physicochemical features and demonstrating tolerance in both in vitro and in vivo conditions. This surfactant possesses one or two lipophilic alkyl chains and two hydrophilic heads. To make bola NIOs, the surfactant is combined with cholesterol and Span 80^®^ in chloroform. The chloroform is subsequently evaporated to yield a thin coating [26].

(i) ***Phytoniosomes (phytoNIOs)***

Numerous plant extracts have been effectively integrated into phytoniosomal formulations with therapeutic efficacy, including *Curcuma longa*, *Narcissus tazetta* L., *Gymnema sylvestre*, *Silybum marianum*, *Myrtus communis* L., *Calendula officinalis*, *Psidium guajava*, *Nerium oleander*, *Justicia adhatoda*, and *Ginkgo biloba* [40,72]. A case study of phytoniosomal formulation is the NIO loaded with extract from *Tradescantia pallida* leaves, developed to provide a prolonged controlled release for antidiabetic treatment. The evaluation of niosomal formulations revealed stable, monodisperse particles with average diameters under 200 nm, increased entrapment efficiency, and regulated drug release patterns extending up to 12 h. The in vitro antidiabetic efficacy of the niosomal formulation demonstrated 89.8% inhibition of alpha-amylase and 88.81% inhibition of hemoglobin, both significantly above the values achieved with acarbose, hence substantiating the formulation’s antidiabetic action. Studies on digestive morphology indicated that the degradation rate of starch and glucose was slower with the formulation than with acarbose. The antidiabetic efficacy of the extract and phytoNIOs was validated by the substantial content of polyphenols [73].

**Figure 2:**
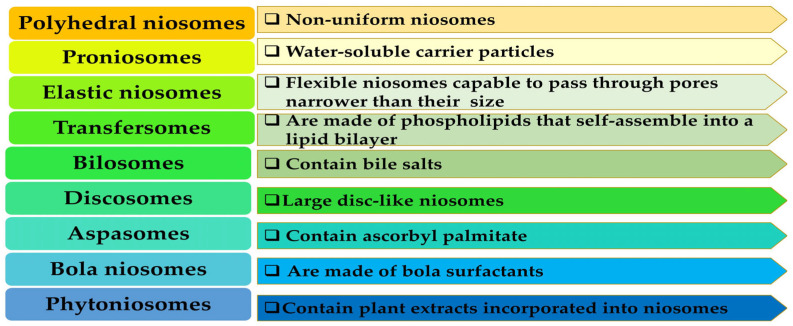
The representation of the main differences between the types of specialized NIO.

Another example of a phytoNIO formulation is the integration of *C. officinalis* extract into an NIO. The effects of phytoNIOs on Vero cells were analyzed in relation to the activities derived from the application of free extract. The results showed that the activity of *C. officinalis* methanolic extract markedly enhanced following its entrapment in a Tween 60 NIO. Consequently, marigold and its encapsulated form within a non-ionic surfactant-based delivery vesicle have significant promise for several bio applications, including its prospective usage as an ingredient in food additives and topical cosmetic compositions [74]. In conclusion, in Figure 2 we depict the main differences between the types of specialized NIO.

## 4. Polysaccharide Niosomal Drug Delivery Systems

**Figure 3:**
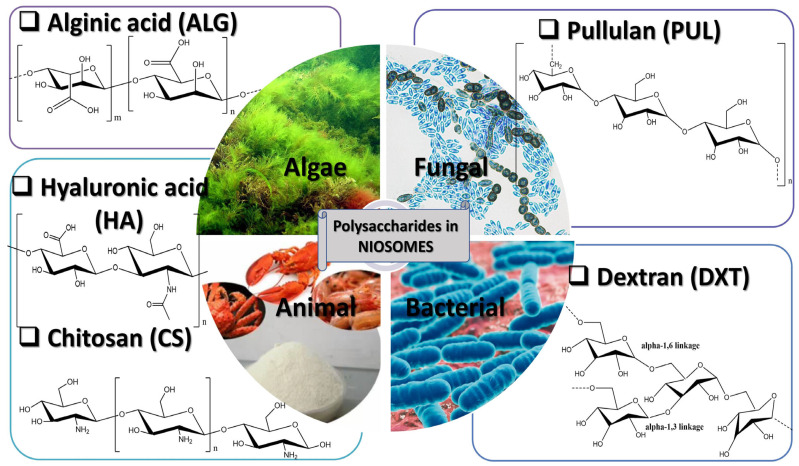
The representation of the main polysaccharides used in NIO formulation.

Different types of polymers can be used in order to design polymeric niosomal drug delivery systems that can have a vast area of application both in the cosmetic and pharmaceutical fields [75]. The polymers can be introduced in order to form a hydrogel that will act as a carrier for the delivery of different active substances incorporated in the niosomal structure [76,77]. Our research work has highlighted the following polysaccharides mainly used in the development of niosomal formulations as DDSs:

✓ derived from algae: alginic acid, alginate (ALG);
✓ of animal origin: chitosan (CS), hyaluronic acid (HA);
✓ of bacterial origin: dextran (DXT); and
✓ of fungal origin: pullulan (PUL) (Figure 3).

### 4.1. Chitosan-Based Niosomal Drug Delivery Systems (CS-DDSs)

Chitosan (CS), a natural polymer, has garnered the interest of pharmacists in recent years, ranking third in worldwide abundance after cellulose. CS consists of many cationic groups, glucopyranose rings, and acetamide/hydroxy groups, which provide the basis for nucleophilic substitution processes [78]). It derives from several sources, including insect exoskeletons and fungal cell walls. This amino polysaccharide is synthesized by the deacetylation of chitin under alkaline circumstances. The notable characteristics of chitosan include its wound healing capabilities, effective mucoadhesion, cholesterol-lowering effects, improvement of membrane permeability, and fungicidal activities. This hydrophilic natural polymer is an excellent contender for tissue engineering and medication delivery systems [79]. CS is non-toxic, biodegradable, and biocompatible, possessing viscous properties and markedly improving medication absorption. These characteristics have resulted in its widespread application in diverse sustained drug delivery systems, such as hydrogels, membranes, nanoparticles, microspheres, and liposomes/NIOs [80].

The CS-DDSs may regulate release rates by precisely adjusting their components, namely CS, NIOs, or their combination. Release rates may be regulated to span from 24 h to over 1320 h, contingent upon the conditions to which NIOs are subjected. Subjecting naked NIOs to tumor-like circumstances can lead to complete release after 144 h, but including CS into the system facilitated a controlled release exceeding 55 days. The release rates may be adjusted by modifying dye concentration and size, CS molecular weight, crosslink density, and packing density [81]. Williams et al. reported that the optimal regulated release was achieved using medium molecular-weight chitosan with a crosslinking ratio of 4:1 (b-glycerophosphate/CS) and a packing ratio of 0.35:1 (NIO/CS). This design method, owing to its adaptability to specific requirements, may be applied to a diverse range of uses, including the administration of labile medicines and targeted drug delivery for different types of tumors or other medical conditions [81,82].

#### 4.1.1. DDSs for Cancer Therapy

Parvathi et al. described the straightforward development of a Fe_3_O_4_ NPs core–shell nano niosome formulation encapsulating ciprofloxacin (CIF) using CS, effectively accomplished without the need of cholesterol or surfactants. The extract of *Garcinia mangostana* fruit peel was utilized to produce biogenic Fe_3_O_4_ nanoparticles (NPs). Fe_3_O_4_ @FA-CS-CIF-NPs nano-NIO were synthesized by concurrently altering Fe_3_O_4_ NPs with CS and folic acid (FA), while incorporating CIF via supramolecular interactions for targeted administration and controlled delivery of the antitumoral agent. The Fe_3_O_4_ @FA-CS-CIF-NPs nano-NIOs had a reactive oxygen species-responsive drug release effectiveness of 93.55% in a malignant cell pH milieu and displayed significant cytotoxicity in vitro against human cervical cancer (HeLa S3) cells, with an IC_50_ of 4.7 μg/mL. The efficacy of Fe_3_O_4_ @FA-CS-CIF-NPs nano-NIOs in suppressing colony formation of HeLa S3 cells and causing S phase cell cycle arrest was evidenced by thorough colony formation and cell cycle investigation. The results indicated a viable strategy for the advancement of tailored cancer treatment with nano-NIOs [17].

5-fluorouracil (5-FU)-loaded niosomal NPs were effectively synthesized, coated with CS, and later crosslinked with tripolyphosphate (TPP) to create niosomal nanogels. CS-coated and TPP-crosslinked NIOs demonstrated a marginal reduction in particle size and a transition in zeta potential from negative to positive values. Furthermore, it was observed that a reduction in the in vitro release rate of 5-FU was accomplished within 72 h by the utilization of CS-coated formulations. All produced formulations demonstrated hemocompatibility in the hemolysis experiment, exhibiting < 5% hemolysis at their maximum doses: 500 µM and 1 mM. The 3-[4,5-dimethylthiazol-2-yl]-2,5 diphenyl tetrazolium bromide (MTT) experiment demonstrated superior anticancer efficacy against B16F10 malignant cells and reduced cytotoxicity towards NIH3T3 normal cells compared to the pure 5-FU within the examined dose range: 10–100 µM. The examination of the cell migration inhibitory characteristics of the formulated NIOs demonstrated comparable outcomes with the in vitro cell viability experiment, showing a greater migration inhibition rate for B16F10 cells compared to free 5-FU, controls, and NIH3T3 cells [83].

Wiranowska et al. evaluated the extracellular and intracellular location of a targeted DDS utilizing paclitaxel (PTX)-encapsulated NIOs incorporated within a CS hydrogel, which exhibits an affinity for MUC1 mucin surface antigen overexpressed on tumor cells OV2008. The findings demonstrate a significantly elevated fluorescence intensity of CS–NIO–PTX next to the OV2008 cell surface in contrast to the normal IMCC3 cell surface. Moreover, intracellular fluorescence intensity was double that of normal IMCC3 cells [84]). A study by Miatmoko et al. showed that incorporating CS into ursolic acid-loaded NIOs (UA-NIOs) enhanced cellular uptake by HeLa cells, which is associated with clathrin-mediated endocytosis facilitating the transport of UA-NIOs into the cells [6].

Another study revealed a pH-responsive nano-niosomal emulsion for sustained release from a CS-based nanocarrier as a viable solution to overcome limitations in cancer treatment using curcumin (CUR). The integration of montmorillonite nanoparticles into nanocomposites enhances loading effectiveness to 76% and facilitates pH-responsive delivery through interactions with hydrogel polymers. The incorporation of nanoparticles into the hydrogel matrix facilitates liaisons between CUR and the polymers, enabling the retention of CUR at pH 7.4 and its release under acidic circumstances, characteristic of cancer cells. Moreover, the nanocomposites’ encapsulation within NIOs enhanced the release duration due to the lipid phase of the nano-niosomal emulsion, hence prolonging the release time. Furthermore, nanocarriers produced by this approach enhanced the apoptotic activity of CUR and decreased the viability of MCF-7 cancer cells compared to cells treated just with CUR [85]. An optimal formulation of CS-coated NIOs for the co-delivery of doxorubicin and vincristine for breast cancer treatment was reported. The coated NIOs exhibited a reduced IC_50_ in the SKBR3 cell line compared to the non-coated NIOs [86].

A different study presents the development of an innovative finasteride (FIN) release mechanism utilizing CS-based nano-NIOs (CS-NIOs) through the thin-film hydration technique. For 10% FIN weight in relation to the total weight of CS-NIO particles (*w*/*w*), the drug encapsulation efficacy in the CS-NIOs was 84.3 ± 3.5%. According to the in vitro release experiments, the medication was released at a rate of 10% on the first day and 70% on the tenth day following drug loading in the micellar/NIO nanoformulation. Density functional theory (DFT) indicates that FIN interacts with the carrier combination with a binding energy of roughly −1.95 eV. Molecular dynamics simulations reveal that FIN is enveloped inside the carrier complex after one nanosecond. Therefore, the CS-NIO nano-formulation demonstrates a promising delivery method for FIN medicines with promise for prostate cancer prophylaxis [87]). Table 3 summarizes the CS-based nano-niosomic formulations investigated for different types of cancer, highlighting the cell lines tested.

#### 4.1.2. DDSs with Antibacterial or Anti-Inflammatory Properties

The production, characterization, and antibiofilm/antibacterial activity of a nanocomposite comprising inorganic NPs (namely, silver or zinc oxide: AgNPs/ZnONPs) and sultamicillin tosylate (ST) co-loaded into NIOs were described by Ashkezari et al. Multiple nanocomposite formulations were developed and subsequently encased with CMC (carboxymethyl chitosan) to augment antibacterial and antibiofilm properties. The remarkable stability of the produced nanocomposite for up to 30 days demonstrates its potential for future medicinal uses. The synergistic effect of inorganic NPs and ST on the antibiofilm/antibacterial properties of the nanocomposite was evaluated against *Staphylococcus aureus*, *Escherichia coli*, *Klebsiella pneumoniae*, and *Pseudomonas aeruginosa*, where a lower dose requirement was observed. Furthermore, the MTT test was used to assess the cytotoxicity of the engineered nanocomposite on the human foreskin fibroblast (HFF) cell line. The findings demonstrated a value of the cell viability > 90% at all evaluated doses in HFF cells. This work demonstrates that the synergistic action of ST and inorganic NPs encapsulated in NIOs combined with CMC hydrogel provides an effective method to augment the antibacterial and antibiofilm properties of drugs, showcasing significant promise for biomedical applications. Therefore, the formulated nanocomposite niosomal system is appropriate for the targeted delivery of hazardous inorganic NPs and pharmaceuticals, and has significant promise as a coating for endotracheal tubes to function as an antibiofilm/antibacterial agent [88].

Regarding the development of NIOs incorporating substances with anti-inflammatory action, the following study was reported. A series of thiolated chitosan (TCS) hydrogels with crocin-loaded NIOs was effectively formulated for aphthous stomatitis (crocin is a carotenoid that is present in saffron flowers’ stigmas—*Crocus sativus* L.). The improved NIO formulation derived from cholesterol and Tween 60 at a 15:85 weight ratio and an L/D ratio of 10 demonstrated a superior EE% value and an adequate particle size. The formulated preparation demonstrated a porous structure, pseudoplastic characteristics, appropriate swelling, improved mucoadhesive qualities, and prolonged release of crocin. Furthermore, the hydrogels incorporating crocin-loaded NIOs yielded favorable results in the model of aphthous stomatitis in animals by enhancing ulcer healing, diminishing the expression of TNF-α and p53, and elevating the production of vascular endothelial growth factor (VEGF) and alpha-smooth muscle actin (α-SMA). The results indicate that NIO-embedded TCS hydrogels provide a viable crocin delivery technology with significant potential for future use in aphthous stomatitis [89].

#### 4.1.3. Gene DDSs

The investigation of lipid–biopolymer hybrid systems has created new opportunities for improving nucleic acid delivery in gene therapy. CS-N-arginine (CS-NA), owing to its enhanced hydrophilicity and intracellular permeability from arginine conjugation, shows potential in these formulations. Chitosomes made of DOTAP/DOPE (1,2-dioleoyl-3-trimethylammonium-propane/1,2-dioleoyl-sn-glycero-3-phosphoethanolamine) and CS-N-arginine derivatives showed variations in their interactions with plasmid DNA, as indicated by Isothermal Titration Calorimetry (ITC) and fetal bovine serum (FBS) protection at a 1:4 ratio. The research indicates that a heightened level of CS-NA substitution significantly influences the complexation of chitosomes with genetic material. Chitosomes exhibited high transfection efficiency in HeLa cells; however, the extent of CS-NA replacement did not seem to affect this efficiency. These findings elucidate the role of CS-NA in a chitosome formulation, and comprehending the function of each component in a hybrid system is essential for the advancement of novel and effective genetic material delivery methods [90].

#### 4.1.4. Nose-to-Brain DDSs

The size range is a crucial feature to consider in the development of nose-to-brain delivery systems, since nano-carriers smaller than 300 nm may be efficiently carried transcellularly via olfactory neurons to the brain. The polydispersion index (PDI) is a crucial measure for assessing the non-uniformity of particle size distribution.

A recent study reported the preparation and in vitro characterization of chitosan-coated NIOs for the nasal transport of clonazepam to the brain. The successful formulation of safe, stable, and efficient nanocarriers necessitates the creation of homogeneous (monodisperse) populations of a certain size. Drug release experiments demonstrated enhanced drug solubility resulting from particle size reduction and a controlled release of 50% of the encapsulated medication. Toxicity experiments conducted using Caco-2 cells demonstrated that the higher dilutions (1:50 and 1:100) exhibited nearly identical cell viability (100%) compared to the untreated control group, demonstrating an absence of cytotoxicity [91].

A separate study detailed the manufacturing of NIOs loaded with lacosamide (LCA) and coated with chitosan (LCA-CS-NIOs) via the Box–Behnken design and thin-film hydration technique to enhance the LCA’s bioavailability, physical stability, and residence time on the nasal mucosa. The influence of three independent variables (cholesterol quantity, CS concentration, and Span 60 quantity) on cumulative release (8 h), entrapment efficiency (EE%), vesicle size, and zeta potential was examined. In comparison to the LCA solution, LCA-CS-NIOs exhibited a prolonged release profile, augmented bioavailability, a twofold enhancement in nasal diffusion and superior brain distribution following intranasal delivery. The study suggested that LCA-CS-NIO nasal drops may be used as a nanometric technology for the management of partial-onset seizures [92].

#### 4.1.5. Oral DDSs

Fayed et al. reported the formulation of NIOs using cholesterol, dicetyl-phosphate, and Span 60, thereafter coated with CS, crosslinked with tripolyphosphate (TPP), and loaded with atorvastatin. The antihyperlipidemic efficacy of the NIOs loaded with atorvastatin was evaluated following oral treatment to hyperlipidemic mice. Equally the CS-encased and non-encased vesicles exhibited a spherical morphology. CS-encapsulated NIOs exhibited a markedly reduced particle size of 96.9 nm in comparison to unencapsulated NIOs (143.2 nm). The obtained values for entrapment efficiency were 57% for CS-encased NIOs and 55.5% for non-encased NIOs, concurrently exhibiting comparable release patterns. The histological and biochemical assessments of the therapeutic effectiveness of several atorvastatin formulations in an in vivo hyperlipidemia animal model indicated that the antihyperlipidemic effects might be prioritized as follows: CS-encapsulated NIOs > non-encased NIOs > drug suspension. The CS coating of NIOs demonstrated promising outcomes in niosomal encapsulation of atorvastatin where it improved the oral bioavailability of the medication, as depicted by the increased cholesterol reduction efficacy [93].

#### 4.1.6. Transdermal DDSs

A research indicated the production of moxifloxacin NIOs (MOX-NIOs) by the passive technique (traditional thin-film hydration process), demonstrating elevated encapsulation efficiency values. Antimicrobial assays demonstrated that niosomal encapsulation significantly improved efficacy against *P. aeruginosa*, whereas integration into CS gel was more crucial for anti-*S. aureus* activity. The high drug-loading capacity, prolonged and constant drug release, together with bioadhesive properties ensure extended local retention and enhanced efficacy; therefore, CS gel-embedded MOX-NIOs presented a promising and effective platform for controlled topical delivery of antimicrobial agents [94].

A work from 2022 reported the use of electrospraying of cefazolin-loaded NIOs over a CS membrane for wound healing purposes. To do this, NIOs were produced using the thin-film hydration technique, followed by electrospinning to create nanofibrous mats. The scaffold was characterized in vitro to assess its physicochemical and biological features. Ultimately, in vivo investigations were conducted to assess the membrane’s potential application in skin regeneration. In vitro findings demonstrated the antibacterial efficacy of the membrane against *S. aureus* and *P. aeruginosa* attributed to the sustained release of cefazolin from the NIOs. The scaffolds exhibited little cytotoxicity. In vivo investigations further validated the membrane’s capacity to promote skin regeneration by facilitating re-epithelialization, tissue remodeling, and angiogenesis. This investigation may demonstrate the potential efficacy of the developed scaffold in skin regeneration and the eradication of bacterial infections [95].

#### 4.1.7. Ocular DDSs

A recent study from 2024 sought to enhance the antifungal efficacy of itraconazole (ITZ) by encapsulating it in bioadhesive NIOs and subsequently coating it with CS. This method is deemed to improve the ocular absorption of ITZ, hence increasing its effectiveness against fungal infections. Moreover, the NIOs were subsequently integrated into pH-sensitive gels formed in situ. This dual method was implemented to enhance the ITZ’s corneal permeation, hence improving the therapy of ocular fungal infections. The NIOs were initially generated by hydrating proNIOs composed of Span 60, cholesterol, and phospholipid. Moreover, in situ gels using NIOs exhibited superior gelling ability and viscosity. The ITZ NIOs coated with CS-laden in situ gels exhibited the maximum ex vivo ocular permeability of ITZ, along with antifungal efficacy and a safe profile. The findings suggest that this formulation offers a viable approach to improve the ocular administration of ITZ, consequently aiding in the treatment of ocular fungal infections [96]. Next, in Table 4 we will present the main characteristics of CS-based niosomes as NIO-CS-DDS.

### 4.2. Hyaluronic Acid-Based Niosomal Drug Delivery Systems (HA-DDSs)

Hyaluronic acid (HA), an essential polysaccharide found in connective tissues, consists of N-acetyl-D-glucosamine and D-glucuronic acid units connected by β-1,3 and β-1,4 linkages [97]. HA comes in several molecular weight forms, each exhibiting unique biological effects: low molecular-weight HA facilitates angiogenesis, whereas high molecular-weight variants inhibit it. HA is a high molecular-weight substance that attracts water, providing hydration, lubrication, and shock absorption, which are vital for cellular development and cosmetic uses [98].

#### 4.2.1. DDSs for Cancer Therapy

Regarding the selection of HA as a polymer for the development of HA-DDSs for cancer therapy, it was reported that HA selectively binds to the cell surface adhesion receptor CD44 which is strongly expressed in many types of cancer and enhances the intracellular transport of anticancer drugs to numerous malignant cells, including breast cancer cells; hence, HA-containing systems can serve as advantageous carriers for successful chemotherapy [99].

An investigation published in 2025 reported the preparation of NIOs coated with HA containing alpha-terpineol (α-TN-HA-NIOs) and evaluated their inhibitory potential against cancer cells. α-TN-HA-NIOs were analyzed utilizing several techniques, including flow cytometry, MTT, and real-time quantitative PCR (RT-qPCR). The results indicate that α-TN-HA-NIOs displayed an encapsulation efficiency (EE) of 89.05%, a spherical shape with a diameter of 279.3 nm, a PDI of 0.30, and a zeta potential of −38.6 mV. α-TN-HA-NIOs showed more cytotoxicity against cancer cells compared to normal HFF cells, as assessed by the cytotoxicity assay. Induction of apoptosis in treated cells was evidenced by the elevated expression of the Bax, p21, and p53 genes, the rise in apoptotic cells, and the stopping of the SubG1 phase. The findings indicate that the nanoformulation developed in this work may be beneficial for cancer treatment [100].

A practical nanosized transdermal DDS for tumor treatment was developed by constructing an amphiphilic HA-based NIO, integrating transdermal and tumor-targeting capabilities inside a single entity. HA esterified with glycerol-monostearate (GMS), referred to as HA–GMS, self-assembled onto the NIO surface, resulting in the formation of an HA–NIO. The multiple layer vesicle exhibited a tiny size (about 40 nm), commendable stability, effective drug encapsulation efficiency, and compatibility with blood. It demonstrated higher endocytosis in mouse breast tumor cells (4T1) compared to the control chitosan nanoparticle, as confirmed both subjectively and quantitatively. The efficacy of HA–NIO skin penetration was demonstrated by an in vivo fluorescence measurement and in vitro stratum corneum model. The histological section analysis validated the safety and efficacy of transdermal permeation. The results demonstrate that the HA–NIO is both intriguing and encouraging for tumor treatment via transdermal delivery [101].

An improved system of niosomal nanoparticles loaded with epirubicin (Epi) and coated with HA (Epi-NIO-HA) has been developed for the targeting treatment of breast cancer cells. The release of Epi from the Epi-NIO-HA exhibited a decrease of 20% in a neutral buffer and of 21% in an acidic buffer compared to the non-embedded category (Epi-NIOs). The apoptosis and cytotoxicity findings for SkBr3 and 4T1 cells demonstrated an approximate twofold enhancement in the Epi-NIO-HA nanoformulation compared to Epi-NIOs and free Epi, hence affirming the high potential of the designed nanocarriers. Furthermore, real-time PCR results indicated the downregulation of cyclin E, cyclin D, and matrix metalloproteinases (MMP-2 and MMP-9) gene expression, and the upregulation of caspase-9 and caspase-3 gene expression. Flow cytometry and confocal microscopy investigations revealed that the cellular absorption mechanism of the Epi-NIO-HA was CD44-mediated. Moreover, in vivo experiments showed that Epi-NIO-HA reduced by 28% the breast tumor volume in mice in comparison with Epi, without adverse effects on the kidneys and liver. The findings demonstrated that HA-functionalized NIOs serve as a viable nanoplatform for the efficient and targeted administration of Epi in the possible treatment of breast cancer [102].

For enhancing the therapeutic efficacy through targeted delivery, a study reported the formulation of HA-coated NIOs encapsulating curcumin (CUR) (CUR-HA-NIOs). CUR-HA-NIOs were synthesized using the thin-film hydration technique and they possessed a size of 142.93 ± 39.71 nm and a charge of −20.67 ± 1.10, demonstrating commendable stability. The encapsulation efficiency was reported as 93 ± 4.35% and a pH-independent release profile was exhibited. CUR-HA-NIOs exhibited a twofold enhancement in antioxidant activity relative to free CUR. CUR-HA-NIOs exhibited superior cytotoxic activity against the MCF-7 cell line, with an IC_50_ of 1.851 ± 0.45 μM, in contrast to free CUR, which had an IC_50_ of 5.230 ± 0.85 μM, representing an augmentation of roughly thrice. These findings suggest that CUR-HA-NIOs may serve as a potential specific nanocarrier to provide targeted bioactivity against cancer, particularly when oxidative stress has a significant adverse consequence [3].

A recent study demonstrated doxorubicin (DOX) encapsulation in cationic NIOs (DOX−NIOs) using the thin-film hydration technique. The DOX−NIO was subsequently modified by electrostatic interaction with HA resulting in a DOX−HA−NIO. The DOX−HA−NIO and DOX−NIO exhibited particle sizes of 182.9 ± 2.3 nm and 120.0 ± 1.02 nm, and zeta potentials of−15.6 ± 0.25 mV and +35.5 ± 0.15 mV, respectively, with a PDI < 0.3. The DOX−HA−NIO exhibited remarkable stability in size and charge over a four-week period at 4 °C and preserved its integrity post-lyophilization. The HPLC findings indicated a DOX encapsulation efficiency of 94.1 ± 4.2%, demonstrating successful entrapment and a gradual, sustained release of DOX over a period of 48 h. The cell viability experiment of DOX−HA−NIO indicated micromolar IC_50_ values against CD44-negative cell lines (NIH/3T3) and an IC_50_ of 14.26 nM against the MCF-7 cell line. In conclusion, the DOX−HA−NIO has been demonstrated to be an efficacious, targeted nanocarrier for DOX in relation to the MCF-7 cell line [103].

#### 4.2.2. Ocular DDS

A group of researchers reported the development of HA-modified cationic NIOs (HA-C-NIOs) aimed at targeted gene transport and effective gene transfection in retinal pigment epithelium (RPE) cells. The cationic NIOs consisting of Tween 80, squalene, and 1,2-dioleoyl-3-trimethylammonium propane (DOTAP) were produced using the ethanol injection technique. Subsequently, HA was incorporated into cationic NIOs to create HA-C-NIOs. The cellular uptake and transfection processes were examined in ARPE-19 cells. The effectiveness of in vivo pEGFP (plasmid-enhanced green fluorescence protein) transfection was assessed in rats. Twenty percent HA-C-NIOs measured around 180 nm in diameter, had a zeta potential of −30 mV, and displayed a spherical morphology in transmission electron microscopy (TEM). The group of HA-C-NIOs with 20% HA alteration had a transfection efficiency that was twice as great. No toxicity was seen in NIO formulations. In vivo assessment in Sprague Dawley (SD) rats showed that HA-C-NIOs may selectively target the retinal layer. The pEGFP-loaded HA-C-NIOs have a gene transfection rate that is six to 6.5 times greater than that of blank pEGFP. Consequently, HA-C-NIOs may offer a viable gene delivery mechanism for effective retinal gene therapy [104].

Utilizing a synergistic approach of NIOs and mucoadhesive HA, Zeng et al. documented the development of an ocular delivery system for tacrolimus (TCLM-HA-NIOs) that utilizes the advantages of both NIOs and HA to enhance ophthalmic bioavailability synergistically. The mucoadhesion of TCLM-HA-NIOs to mucin was examined using surface plasmon resonance, in contrast to non-coated NIOs and HA solution. The findings indicated that NIOs exhibited adherence to mucin, and the HA covering enhanced this attachment. In vivo precorneal retention was assessed in rabbits, revealing that HA-coated NIOs considerably extended the residence time of TCLM compared to non-coated NIOs or solution. The pharmacokinetics test of watery humor indicated that the area under the curve for HA-coated NIOs was 1.2 times and 2.3 times more than that of non-coated NIOs and of the TCLM suspension, respectively. Furthermore, the synergistic improvement of corneal permeability by the hybrid delivery method on TCLM was seen and validated using a confocal laser scanning microscope. The findings demonstrated that the hybrid method enhanced TCLM ocular administration through watery humor pharmacokinetics, mucoadhesion, transcorneal permeability, and precorneal retention. Consequently, HA-coated NIOs may provide a potential method for ocular targeted administration of TCLM [13].

#### 4.2.3. Transdermal DDSs

Li et al. developed an HA-modified steareth-2-based niosomal formulation (HA-NIO) exhibiting targeting capabilities and remarkable deformability for the delivery of ergothioneine (EGT) to combat skin damage induced by UV exposure. EGT is a compelling mitochondrially targeted antioxidant featuring a distinct absorption mechanism. Its distinctive composition enables the EGT-HA-NIO to demonstrate significant mechanical softness, enabling its deformation to traverse the stratum corneum via the intercellular space without causing rupture. HA alteration enhances EGT’s ability to target human dermal fibroblasts (HDFs) for intracellular delivery, facilitating more mitochondrial distribution without reliance on the specialized EGT transporter OCTN-1 (organic cation transporter 1). Leveraging the aforementioned features, a sufficient quantity of active EGT was concentrated in the targeted cellular locations, mitigating the formation of ROS (reactive oxygen species) generated by UV radiation, the release of inflammatory factors, DNA damage, and mitochondrial dysfunction. The findings from the in vivo experiment indicated that EGT-HA-NIO may markedly restore epidermal thickness and shape to normal levels, reduce collagen degradation, and efficiently avert UV-induced skin damage. HA-NIO’s capacity to traverse biological barriers and administer pharmaceuticals may facilitate the advancement of suboptimal drug penetration therapies for conditions such as dermatological disorders, malignancies, and bacterial infections [105].

#### 4.2.4. Pulmonary DDSs

The ethanol injection technique was employed to create NIOs with cholesterol as the lipid and Span 80 as the non-ionic surfactant. HA-loaded NIOs exhibited a particle size of 177.6 nm and an entrapment efficiency (EE%) of 95%. The drug release trials from the HA-loaded NIOs demonstrated a regulated release profile lasting up to four days. The HA-loaded NIOs exhibited robust stability at both room temperature (25–30 °C) and refrigerated conditions (4–8 °C). The DPPH (2,2-diphenyl-1-picrylhydrazyl) antioxidant experiment demonstrated superior enzyme inhibition compared to the free HA solution. In vivo pharmacokinetic drug assessment parameters indicated a higher plasma concentration of HA compared to free HA solution administered via the aerosol method. Furthermore, organ biodistribution studies indicated a higher localization of HA in the lungs compared to other organs [106].

#### 4.2.5. Cardiac DDSs

A recent study from 2024 described the formulation of NIOs including β-sitosterol (BetaS), employing Tween 80 as the stabilizer and cholesterol as the lipid, and coated with HA to improve the specificity and effectiveness of β-sitosterol in cardiac tissue. The niosomal formulation produced was spherical, measuring around 158.51 ± 0.57 nm, with a drug loading of 8.07 ± 1.62% and an entrapment efficiency of 93.56 ± 1.48%. Cytotoxicity was assessed in H9c2 cardiac cells by the MTT test. Intravenous administration of BetaS-HA-NIOs at a dosage of 10 mg/kg showed a substantial reduction in cardiac troponin-I (cTn-I), lipid peroxidation (MDA), lactate dehydrogenase (LDH), aspartate aminotransferase (AST), and creatine kinase-MB (CK-MB) levels. Tissue histology revealed a significant capacity for cardiac tissue regeneration following treatment with BetaS-HA-NIOs, as well as robust cardioprotection against myocardial damage produced by isoproterenol in Sprague Dawley rats. Therefore, improving the therapeutic efficacy of BetaS by NIO surface modification shows potential for alleviating cardiac injury caused by cardiotoxicity [15]. Next, in Table 5 we will present the main characteristics of HA-based niosomes as NIO-HA-DDS.

### 4.3. Alginate-Based Niosomal Drug Delivery Systems (ALG-DDSs)

Algal polysaccharides have physiological characteristics such as antioxidant, anti-inflammatory, anticancer, and immune-regulating capabilities [52]. Alginate (ALG) is a biodegradable polysaccharide and a negatively charged polymer derived from brown seaweed or metabolic byproducts of *Azotobacter vinelandii* and *Pseudomonas* spp. bacteria. The structure comprises two sterically distinct repeating units: β-mannuronic acid (M) and α-l-glucuronic acid (G) connected via 1,4 bonds in various amounts. The negative charge originates from the carboxyl groups located on the ring scaffold of both M and G monomers. Consequently, due to the stability of ALG and its pH sensitivity, formulations for sustained or regulated drug delivery systems utilizing alginates have been documented [19]. The distinctive characteristics of sodium alginate, including its excellent tissue compatibility, non-toxicity, biodegradability, hydrophilicity, and affordability, render it appropriate for applications in tissue engineering, particularly in skin regeneration and the treatment of exuding wounds with an improved healing process. ALG dressings are extensively utilized in wound management because to their healing and hemostatic characteristics, as well as their ability to absorb exudate [107] Among polymer hydrogels, alginate is an appropriate substrate for NIO loading due to its biocompatibility, biodegradability, cell adhesion properties, and cost-effectiveness [108].

#### 4.3.1. DDSs for Cancer Therapy

The utilization of algal polysaccharides as carriers for chemotherapy drugs presents two primary advantages: firstly, the three-dimensional network structure of the formulation facilitates precise drug loading and tumor-specific environmental responsiveness, enabling controlled release via physical embedding or chemical bonding, thereby markedly enhancing drug accumulation efficiency at tumor sites. Secondly, the biological actions of algal polysaccharides, including immunomodulatory and anti-angiogenic properties, can exert synergistic anticancer effects in conjunction with chemotherapeutic agents. The “carrier-as-drug” characteristic overcomes the constraints of conventional synthetic materials as delivery mechanisms, and its natural origin significantly mitigates the possible biological toxicity associated with synthetic nanocarriers [109]).

An innovative ALG-DDS utilizing 3D-printed gelatin-alginate scaffolds embedded with NIOs loaded with paclitaxel (PTX-GT_ALG-NIOs) was reported by Hosseini et al. PTX-GT_ALG-NIOs and PTX-NIOs exhibited prolonged drug release and biodegradability. Cytotoxicity investigations demonstrated that the engineered PTX-GT_ALG-NIO scaffold exhibited less than 5% cytotoxicity towards the MCF-10A non-tumorigenic breast cell line while displaying 80% cytotoxicity against MCF-7 breast cancer cells, far surpassing the anti-cancer efficacy of control specimens. In the migration examination (scratch assay), a decrease of roughly 70% in the covered surface area was observed. The anticancer efficacy of the engineered nanocarrier can be ascribed to the modulation of gene expression, evidenced by a substantial upregulation in the expression and action of pro-apoptotic genes (CASP-9, CASP-8, and CASP-3) and of metastasis-inhibiting genes (p53 and Bax), along with a notable downregulation of metastasis-promoting genes (MMP-2, MMP-9, and Bcl2). Furthermore, flow cytometry studies indicated that PTX-GT_ALG-NIOs significantly decreased necrosis and enhanced apoptosis [110].

Another study reported the utilization of 3D-printed gelatin–alginate nanocomposites including NIOs loaded with doxorubicin (DOX-GT_ALG-NIOs) as a sophisticated pH-sensitive drug delivery method. The results indicated that the synthesized NIO possesses a spherical morphology with a size ranging from 60 to 80 nm. DOX-GT_ALG-NIOs and DOX-NIOs exhibited sustained drug release and biodegradability. The cytotoxicity analysis demonstrated that the developed DOX-GT_ALG-NIO scaffold exhibited 90% cytotoxicity against MCF-7breast cancer cells while showing less than 5% cytotoxicity against the MCF-10A non-tumor breast cell line, significantly surpassing the antitumor effectiveness of the control specimens. The scratch assay, utilized as an indication of cell movement, revealed a nearly 60% decrease in the covered surface area. Gene expression may elucidate the antitumor effects of engineered nanocarriers, which markedly diminished the expression of metastasis-promoting genes (MMP-2, MMP-9, and Bcl2) and substantially increased the expression and activity of pro-apoptotic genes (CASP-9, CASP-8, and CASP-3). Significant suppression of metastasis-related genes (p53 and Bax) was noted. Furthermore, flow cytometry findings indicated that DOX-GT_ALG-NIOs significantly reduced necrosis and markedly increased apoptosis. These studies mentioned above confirm that using 3D printing with niosomal formulation is a successful technique for producing innovative nanocarriers for successful drug delivery applications [111].

A separate investigation indicated the optimal formulation of DOX_CIS-ALG-NIOs, a NIO-based nanocarrier coated with alginate, designed for the co-delivery of doxorubicin (DOX) and cisplatin (CIS) in the therapy of ovarian and breast malignancies, aiming to reduce medication dosages and address multidrug resistance. DOX_CIS-ALG-NIOs exhibited an efficacy of encapsulation of 80.65 ± 1.80% for DOX and 65.54 ± 1.25% for CIS. The zeta potential of DOX_CIS-NIO nanocarriers decreased following alginate coating. The MTT experiment indicated that the IC_50_ of DOX_CIS-ALG-NIOs was inferior to that of the DOX_CIS-NIO formulations and the free drugs. Cellular and molecular studies revealed that DOX_CIS-ALG-NIOs significantly enhanced the rate of apoptosis induction and induced cell cycle arrest in A2780 and MCF-7 cancer cells, in comparison to DOX_CIS-NIOs and free medicines. The activity of caspase 3/7 was elevated following treatment with coated NIOs in comparison to uncoated NIOs and the drug-free control. The combined inhibitory effects of DOX and CIS on cell growth were seen in A2780 and MCF-7 cancer cells. All anticancer testing results indicated that the co-delivery of DOX and CIS using ALG-coated niosomal nanocarriers was successful for the treatment of ovarian and breast cancer [112]. 

Akbarzadeh et al. delineate the formulation of NIOs coated with calcium ALG as a nanocarrier encapsulating curcumin (CUR-ALG-NIOs), intended for the eradication of breast cancer cell lines. The aforementioned formulations were administered to breast cancer cell lines MDA-MB231 and SKBR3, and gene expression levels were evaluated to assess the effectiveness of this unique therapeutic method. The formulation enhanced the efficacy of chemotherapy owing to the presence of ALG. The in vitro evaluations demonstrated that the CUR-ALG-NIOs effectively induced significant apoptosis in the examined breast cancer cells, which correlates with the upregulation and downregulation of various gene expressions (i.e., caspase-3, caspase-9, Bcl-2, cyclin E, cyclin D, P53, and Bax). This work presented a novel paradigm for alternative therapeutic strategies, including chemotherapy regimens in breast cancer cells, by advancing the biocompatibility of nanoparticles and improving necessary therapeutic applications [113]

#### 4.3.2. Oral DDSs

Various metformin-loaded niosomal and chitosomal (MTF-NIOs and MTF-CS-NIOs, respectively) formulations were created and appropriately described; nevertheless, they failed to achieve the expected sustained release. The entrapment of both types of colloidal dispersions within calcium ALG beads significantly diminished the drug discharge at the gastric medium (from a maximal level of 30% to 18%) and facilitated a sustained release in simulated intestinal fluid, which was effectively modulated by adjusting the calcium ALG percentage in the beads. In vivo studies on rats demonstrated a notable enhancement of MTF’s hypoglycemic effect when administered orally as chitosomal and, to an even greater extent, as niosomal dispersion encapsulated in ALG beads, compared to both the drug alone and ALG beads containing the unmodified drug. The sustained and intensified therapeutic response over time offered by the drug-in MTF-ALG-NIO formulation may be advantageous for maintaining stable blood glucose levels following oral administration, facilitating a reduction in dosage and associated side effects, while enhancing patient compliance [114]. In Table 6 are presented the main characteristics of ALG-based niosomes as NIO-ALG-DDS.

### 4.4. Pullulan-Based Niosomal Drug Delivery Systems (PUL-DDSs)

Pullulan (PUL) is a water-soluble and neutral homo-polysaccharide consisting of constantly repeating α-(1 → 4)-maltotriosyl units (3-D-glucopyranosyl) linked by α-(1 → 6) connections. It is often produced by the aerobic fermentation of the polymorphic fungus *Aureobasidium pullulans*, resulting in an amorphous slime substance. The molecular mass of this exo-polysaccharide varies from 4.5 × 10^4^ to 6 × 10^5^ Da, contingent upon the environment for yeast cultivation [115]. Due to their spinnability, moldability, non-carcinogenicity, biodegradability, non-toxicity, edibility, non-mutagenicity, biocompatibility, and non-immunogenicity, polysaccharides, particularly pullulan, are selected as the preferred materials for the development of delivery systems for drug delivery and genetic material transfer, possessing the desired biopharmaceutical and therapeutic characteristics [46,116].

#### 4.4.1. Oral DDSs

A 2006 study describes the preparation and assessment of polysaccharide-capped NIOs for their immunostimulant capabilities. Tetanus toxoid (TT) antigen was encapsulated in NIOs by the reverse phase evaporation process and coated with a pullulan derivative, namely O-palmitoyl pullulan (OPP). The serum IgG antibody level was evaluated to determine the immune-stimulating impact of these carriers after oral administration. OPP-capped TT-loaded NIOs were reported to trigger a superior humoral response compared to their unmodified counterparts. Moreover, OPP-encapsulated NIOs elicit an immunological response nearly comparable to that induced by intramuscular administration of alum-adsorbed tetanus toxoid. Mucosal IgA levels indicate that polysaccharide-capped niosomal systems may serve as a viable oral vaccination delivery mechanism [75].

#### 4.4.2. Delivery of Preserving Food Agents

The nanoencapsulation of catechins as proNIOs was successfully described using Span 60 and cholesterol, along with wall materials such as maltodextrin, lactose, and PUL, via the thin-film hydration approach. The proNIOs exhibited prolonged linear release and maintained antioxidant activity under in vitro settings. The minimum particle size was 193.57 nm for PUL-based catechin-loaded proNIOs with an agitation duration of 1.5 h. FTIR and XRD demonstrated the encapsulation of catechins in the form of proNIOs. Pullulan was suggested as the wall material for the nanoencapsulation of catechins in the form of proNIOs. Milk and yogurt enriched with catechin-loaded proNIOs exhibited satisfactory organoleptic quality, with no significant alterations in physicochemical parameters relative to pasteurized milk and yogurt. This study concludes that proNIOs are an effective alternative for enhancing the bioavailability of catechins in food while preserving their intended functional benefits [46].

### 4.5. Dextran-Based Niosomal Drug Delivery Systems (DXT-DDSs)

Dextran (DXT) is an extracellular homopolysaccharide with bacterial origin, characterized by a primary chain of continuous α-(1,6)-linked d-glucopyranose nucleus, with branches mostly consisting of side-chains of α-(1,6) glucose entities connected by α-(1,3) branch connections to the α-(1,6)-linked chains. Certain dextrans possess α-(1,4) or α-(1,2) branch connections, contingent upon the bacterial origin [117]. Furthermore, DXT is acknowledged as a potential substitute for PEG in stealth coating materials and for its capacity to impede opsonization. DXT’s structure has several hydroxyl groups inside its anhydroglucose unit, allowing for chemical modifications that yield unique characteristics. Consequently, careful chemical modification of this therapeutically valuable polysaccharide to produce amphiphilic copolymers, so facilitating self-organization in aquatic environments, is of significant interest for effective drug delivery applications [117,118].

#### 4.5.1. DDSs for Cancer Therapy

Sharma et al. describe the formulation of FITC dextran (FITC-DXT)-loaded NIOs. The NIOs were synthesized using the thin-film hydration technique, which entailed the self-assembly of cholesterol and Tween 80. Encapsulation experiments involving the active compounds curcumin (CUR) and doxorubicin hydrochloride (DOX) showed that CUR concentrated in the shell, whereas DOX was retained in the inner aqueous center of the NIO formulation. Confocal investigations showed that nile red preferentially adsorbs to the head group of Tween 80, resulting in the formation of two distinct layers inside the shell. The NIOs were self-degraded in phosphate-buffered saline (PBS) via a sequential process, resulting in the formation of interconnecting pores, culminating in full degradation after one week. The release profile delineates two phases: (i) an initial release of DOX over the first two days, succeeded by (ii) the release of CUR over a span of seven days. Synergistic cytotoxicity was demonstrated in dual-drug-loaded NIOs against HeLa cell lines. Consequently, these NIOs demonstrate potential as an effective DDS for both hydrophobic and hydrophilic drugs [119].

#### 4.5.2. Oral DDSs of Anti-Diabetic Agents

A further study described the identical film hydration technique, using Tween 80 and cholesterol to generate the niosomal formulation for the simultaneously encapsulation and release of hydrophilic and hydrophobic anti diabetic medications. Confocal laser scanning microscopy (CLSM) studies showed that the hydrophobic molecule represented by the nile red is preferentially concentrated in the shell, whereas the hydrophilic molecule represented by fluorescein isothiocyanate dextran (FITC-DXT,) was retained in the aqueous core of the NIO. The examination of NIOs using Fourier transform infrared (FTIR) spectroscopy demonstrated the creation of hydrogen bonds between cholesterol and Tween 80. The encapsulation efficiencies for metformin hydrochloride (MTF) and glipizide (GLP) were determined to be 58.72% and 67.64%, respectively. The drug release assays conducted in buffers with varying pH levels (simulating blood plasma pH, cellular endosomal, and stomach conditions) demonstrated that the drug release exhibited a linear profile for 8–10 h, thereafter decelerated, and continued for 12–14 h. Consequently, this formulation presents a viable DDS for the combinatorial sustained release of antidiabetic medications [120].

## 5. The Role of Polysaccharides in the Development of Polysaccharide–NIO-Based DDSs

**Figure 4:**
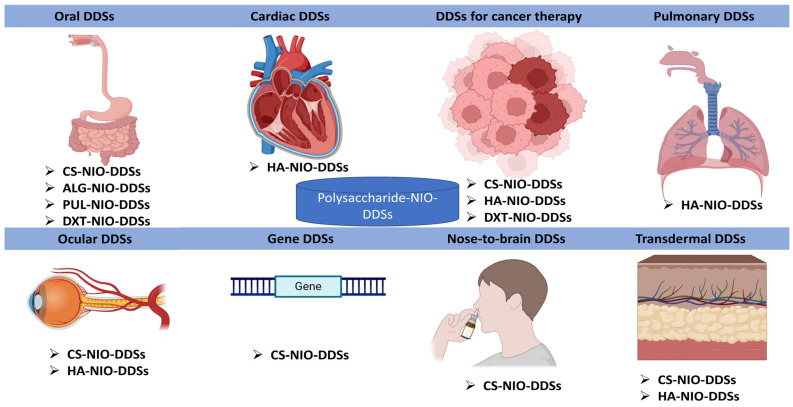
The representation of the main medical applications of polysaccharide–niosomal (NIO) formulations as DDSs (made with the help of BioRender.com).

The development of niosomal nanoformulations based on polysaccharides is a continuously expanding field and enjoys the attention of many researchers in the pharmaceutical and medical fields because it can solve problems related to the bioavailability of active substances incorporated into these nanometric formulations, substances with problematic pharmacokinetic parameters. Thus, numerous DDSs that contained biopolymers from the polysaccharide class and nanoformulations from the niosomal class were reported to have a wide range of applications. Figure 4 serves to exemplify the area of action/therapeutic application of these DDSs that contain NIOs but also the polysaccharides used in their formulation.

NIOs, due to their versatility, can encapsulate numerous active substances, but more innovative systems for systemic NIO formulations and their targeted delivery were necessary since most research until now mainly investigated locally delivered or topical NIOs. Therefore, in order to address this problem, researchers developed the formulation of NIOs coated with various polysaccharides, in order to ensure a controlled release, a targeted release, or a prolonged release, depending on the desired application. Further to highlight the important role of polysaccharides in the formulation of these DDSs, we realized Table 7, which emphasizes the function of polysaccharides in each type of niosomal DDS taken into consideration.

## 6. Current Limitations and Future Perspectives

Despite the polysaccharide-based NIOs’ advantages and efficiency proved in vitro and in preclinical studies performed until now, in order to further pursue their clinical translation and implementation, some barriers should be addressed in the future regarding: reproducible manufacturing and scale-up production especially regarding the choice of appropriate surfactants, pH and temperature conditions, predictive drug-loading, and releasing capacity; stability maintenance and storage limitations [121]( in vivo bioavailability; as well as the absence of standardized protocols for regulatory approval [122].

NIOs’ integrity can be altered by steam or heat sterilization methods. The storage conditions should be refined in order to avoid the current risks of bilayer components’ chemical degradation reactions (oxidation of incorporated drugs, surfactants’ hydrolysis, fusion, aggregation, or drug leakage). New industrial methods should also be applied in order to replace the currently time-consuming sonication, extrusion, or thin-film hydration [4]. Furthermore, new studies are necessary to improve NIOs’ bioavailability by reducing their quick clearance due to phagocytosis, as well as further in vivo studies to explore and diminish the immunogenicity or cytotoxicity potential especially associated with some surfactants [123].

Targeted drug delivery using niosomal systems can be accomplished through two methodologies: (i) passive targeting of the reticuloendothelial system (RES), a component of the immune system consisting of phagocytic cells within reticular connective tissue, and (ii) conjugation with functionalized ligands (polysaccharides) to facilitate targeting to specific organs or tissues [4,5]. We reported in this review that HA-DDSs for cancer therapy are selectively bound to CD44, enhancing drug transport to malignant cells, including breast cancer cells, making them advantageous carriers for successful chemotherapy [99]. NIO-based targeted drug delivery systems hold considerable potential to revolutionize modern cancer therapeutics by enabling precise and localized drug release at tumor locations, hence mitigating the side effects associated with traditional chemotherapy [17].

A future direction to explore is the one regarding specialized polysaccharide-based NIOs that can be integrated with stimuli-responsive carrier gels and/or eutectic/ionic liquids to enhance their retention durations at targeted locations of action. The utilization of specialized NIOs, exhibiting enhanced performance relative to conventional NIOs, seems to be one of the most efficacious methods to maximize the usability of this technology and perhaps accelerate innovation towards the creation of market-ready formulas and products [122].

Other directions to be explored include the use of 3D printing of these niosomal formulations, as a relatively new field, which can be a promising strategy for constructing three-dimensional structures with precise control over scaffold geometry, hence facilitating the reproduction of complex tissue architectures. Further optimization of polymer concentrations to enhance the rheological characteristics of bioinks is a critical topic for continued research [124].

Moreover, clinical studies exploring interpatient response variability due to different disease state, metabolism profile, and immune system reactivity would enable reaching the goal of personalized therapy and cosmetology [121].

## 7. Conclusions

The application of polysaccharide-based DDSs has become more relevant for the treatment of many diseases. Polysaccharides are utilized in the development of DDSs as niosomal formulation to enhance drug concentration in the bloodstream, as well as the biocompatibility and biodegradability of the pharmaceuticals. Moreover, polysaccharides impart unique properties to these composites, including mechanical strength, which enhances the effectiveness of drug administration. Given that NIO vesicles are susceptible to aggregation, drug leakage, and other instability issues during prolonged storage, the incorporation of polysaccharides is an efficient method to enhance their stability. The modification with polymers can increase the pharmacological activities and therapeutic benefits of different insoluble medicines and bioactive compounds by enhancing bioavailability, solubility, and retention duration. Also, polysaccharide-coated NIOs as targeted DDSs provide significant potential to transform contemporary cancer therapies by facilitating precise and localized drug release at tumor sites, hence reducing the adverse effects linked to conventional chemotherapy.

This review research has focused on the many uses of polysaccharides and their derivatives as crucial and beneficial biopolymers for the advancement of NIO-DDSs.

## Figures and Tables

**Table 1:** Specialized types of NIO (after [4,26]).

Types of NIO	Main Characteristics	Reference
***Polyhedral niosomes***	✓ Non-uniform spherical vesicles.	[32]
***Proniosomes***	✓ Made by coating a water-soluble carrier with a slender layer of non-ionic surfactant; compact liquid crystalline niosomal formulation.	[13]
***Elastic niosomes***	✓ Flexible NIOs that may pass through pores narrower than their sizing without losing shape.	[33,34]
***Transfersomes***	✓ Made of phospholipids that self-assemble into a lipid bilayer (edge activator) in an aqueous milieu shaping a vesicle.	[35]
***Bilosomes***	✓ Vesicular system containing bile salts.	[36]
***Discosomes/Discomes***	✓ Large disc-like NIOs.	[37]
***Aspasomes***	✓ Bilayer-forming product that creates vesicles with a negatively charged lipid (dicetyl phosphate), ascorbyl palmitate and cholesterol.	[38]
***Bola niosomes***	✓ NIOs made of bola surfactants (discovered in the early 1980s in archaebacteria membranes).	[26,39]
***Phytoniosomes***	✓ Plant extract incorporation in NIOs.	[40]

**Table 2:** Recent bilosome formulations.

Active Substance	Main Characteristics	Ref.
***Cefepime (CFP)***	✓ Unique oral sustained-release drug delivery method that addresses the challenges of limited oral bioavailability and ↓ half-life of CFP, ↑intestinal permeability, and mitigates adverse side effects.	[57]
***Losartan (LST)***	✓ The renal protective properties of LST were enhanced via encapsulation in bilosomes (↓ serum creatinine, ↓ blood urea nitrogen (BUN), and ↓ protein levels in urine, ↑ efficacy in regulating renal inflammation and fibrosis, as seen by ↓ levels of renal TNF-α, IL-6, NF-κB, and CD45).	[58]
***Itraconazole*** ***(ITZ)***	✓ Bilosomes serve as the delivery route for the specific transport of ITZ, a strong anticancer drug, to hepatic cells. Radiolabeling and biodistribution experiments with radiolabeled itraconazole validated the targeted accumulation of optimized bilosomes in hepatic cells, establishing a mechanistic connection to the observed ↑ in cytotoxicity assays against cell line HepG2.	[59]
***Rizatriptan (RTP)***	✓ When compared to RTP solution delivered intranasally, the RTP’s relative bioavailability following intranasal administration of improved RTP bilosome formulation was 2.94 ± 0.27 times higher, showing ↑ bioavailability. Also, the nose-to-brain drug transport percentage and drug targeting efficiency percentage showed that the RTP-BLSs had ↑ brain targeting and ↑brain targeting efficiency.	[60]
***Sodium tauroursodeoxycholate (TUDCNa)/*** ***oleanoic acid (OA)***	✓ Bilosomes demonstrated ↑ permeability and ↑ oral bioavailability along with a longer intestinal retention period. TUDCNa and OA worked together to produce the greatest therapeutic effect in a model of type 2 diabetes mellitus in mice that was induced by streptozotocin administration. They were able to replace cholesterol in traditional liposomes and offer a novel method for the oral delivery of hypoglycemic medications, which is a novel approach to combination therapy.	[61]
***Curcumin (CUR)***	✓ When doxorubicin-resistant breast cancer (MCF-7/ADR) cell lines were incubated for 48 h, the bilosomes based on d-α-tocopheryl -polyethylene glycol 1000 succinate (TPGS) and incorporated with CUR - demonstrated a strong response, as evidenced by a major decrease in IC_50_ value against multidrug-resistant (MDR) cancers.	[62]
***Venlafaxine (VLF)***	✓ The biodistribution research revealed that the intranasal delivery of VLF-hyaluronic acid (HA) transbilosomes (TBLs) had a relative bioavailability of 441 percent in the brain and 288 percent in plasma when compared to the intranasal administration of VLF solution (VLF-SOL). Furthermore, compared to intravenous VLF-SOL, intranasal distribution of VLF-HA-TBLs showed a much greater bioavailability (512%) in the brain.	[63]

**Table 3:** Chitosan (CS) NIO formulations as DDSs of bioactive agents for cancer therapy.

Bioactive Agent/Active Substances	Formulation	Type of Cancer/Cell Line Tested	Ref.
***biogenic Fe_3_O_4_ nanoparticles (NPs)*** ***Ciprofloxacin (CIF)*** ***Folic acid (FA)***	Fe_3_O_4_ @FA-CS-CIF-NP nano-NIOs.	Cervical cancer/HeLa S3	[17]
***5-Fluorouracil(5-FU)***	CS-coated and TPP-crosslinked 5-FU NIOs.	Skin cancer/B16F10	[83]
***Paclitaxel (PTX)***	PTX NIOs incorporated within a CS hydrogel.	Ovarian cancer/OV2008	[84]
***Curcumin (CUR)*** ***Montmorillonite (MMT)***	CUR-loaded MMT nanoparticles. CS –agarose nano-niosomal emulsion.	Breast cancer/MCF-7	[85]
***Finasteride (FIN)***	CS-based nano-NIOs encapsulated with FIN.	Prostate cancer	[87]

**Table 4:** Summary of NIO-CS-DDS characteristics, preclinical evaluation, and applications.

Type of Niosomal CS-DDS	Formulation Considerations	Preclinical Tests and Findings	Application	Ref.
***Antibacterial/*** ***anti-inflammatory DDS***	Nanocomposite with inorganic nanoparticles and sultamicillin tosylate co-loaded in NIOs coated with carboxymethyl CS.	❖ Antibacterial assays (S. aureus, E. coli, K. pneumonia, P. aeruginosa); ❖ MTT test (human foreskin fibroblast—HFF) with cell viability > 90% at all evaluated doses in HFF cells.	❖ Coating endotracheal tubes to function as an antibiofilm agent.	[88]
***DDS for cancer treatment***	Paclitaxel-encapsulated NIOs incorporated within a CS hydrogel	❖ Affinity for MUC1 mucin surface antigen overexpressed on tumor cells.	❖ Targeted cancer DDSs.	[6]
	pH-responsive nano-niosomal CS emulsion incorporating montmorillonite/ curcumin nanoparticles	❖ ↓↓ viability on MCF-7 cancer cells; ❖ Enabling the retention of curcumin at pH 7.4 and its release under the acidic circumstances, characteristic of cancer cells.	❖ Targeted cancer DDSs.	[85]
***Nose-to-brain DDS***	Chitosan-coated NIOs (thin layer evaporation–paddle stirring) encapsulating clonazepam	❖ Toxicity experiments using Caco-2 cells indicated an absence of cytotoxicity; ❖ Drug release experiments demonstrated ↑↑ drug solubility.	❖ Management of partial-onset seizures.	[91]
***Oral DDS***	NIOs formed from cholesterol, dicetyl-phosphate, and Span 60, coated with CS, crosslinked with tripolyphosphate (TPP), and loaded with atorvastatin	❖ In vivo hyperlipidemia animal model indicated that the antihyperlipidemic effects are CS-encapsulated NIOs+atorvastatin > non-encased NIOs+ atorvastatin > atorvastatin suspension.	❖ DDSs with ↑↑oral bioavailability of statins for ↑↑ efficacy of hyperlipidemia treatment.	[93]
***Transdermal DDS***	Electrospraying of cefazolin-loaded NIOs (thin-film hydration) over a CS membrane	❖ Antibacterial assays (*S. aureus*, *P. aeruginosa*); ❖ Cytotoxicity assay; ❖ In vivo investigations demonstrated the membrane’s capacity to promote skin regeneration; ❖ ↑↑ re-epithelialization, tissue remodeling, and angiogenesis.	❖ Skin regeneration and eradication of bacterial infections.	[95]
***Ocular DDS***	Bioadhesive NIOs (hydrating proNIOs containing Span 60, cholesterol, and phospholipid) encapsulating itraconazol coated with CS and integrated into pH-sensitive gels formed in situ	❖ Exhibited the maximum ex vivo ocular permeability of itraconazol along with antifungal efficacy and a safe profile.	❖ Treatment of ocular fungal infections.	[96]

**Table 5:** Summary of NIO-HA-DDS characteristics, preclinical evaluation, and applications.

Type of Niosomal HA-DDS	Formulation Considerations	Preclinical Tests and Findings	Application	Ref.
***DDS for cancer therapy***	NIOs formed from cholesterol and Span 60 (thin-film hydration) alpha-terpineol-loaded and coated with HA.	❖ ↑↑ cytotoxic effects on cancer cells than normal human foreskin fibroblast (HFF) cells due to their ability to target CD44-positive PANC-1 gastric cells.	❖ Tumor-targeting strategy.	[100]
	HA esterified with glycerol-monostearate self-assembled onto small-sized multilamellar NIOs.	❖ In vivo fluorescence measurement and in vitro stratum corneum model demonstrated the safety and efficacy of transdermal permeation.	❖ Tumor treatment via transdermal delivery.	[101]
	HA-coated NIOs encapsulating curcumin (thin-film hydration).	❖ ↑↑ antioxidant activity compared with curcumin; ❖ ↑↑ cytotoxic activity against the MCF-7 cell line compared with curcumin.	❖ Targeted bioactivity against cancer, with ↑↑ oxidative stress.	[3]
	Doxorubicin-loaded coationic NIOs (thin-film hydration) subsequently modified by electrostatic interaction with HA.	❖ Cell viability experiment showed micromolar IC_50_ values against CD44-negative cell lines.	❖ Targeted nanocarrier for doxorubicin in relation to the MCF-7 cell line.	[103]
***Ocular DDS***	HA-coated cationic NIOs (Tween 80, squalene, and 1,2-dioleoyl-3-trimethylammonium propane) produced by ethanol injection technique.	❖ In vivo assessment in Sprague Dawley rats indicated that it may selectively target the retinal layer; ❖ Effectiveness of in vivo pEGFP (plasmid-enhanced green fluorescence protein) transfection was assessed in rats, where it was ↑↑ than blank pEGFP.	❖ Viable gene delivery mechanism for effective retinal gene therapy.	[104]
	HA-coated NIOs (poloxamer 188, soybean phosphatidylcholine, and cholesterol) produced by reconstituting the proNIOs and loaded with tacrolimus.	❖ ↑↑ adhesion strength and rate of NIO to mucin; ❖ ↑↑ tacrolimus’s precorneal retention in vivo, ↑↑ its aqueous humor pharmacokinetics.	❖ Targeted ocular delivery of tacrolimus.	[13]
***Transdermal DDS***	Ergothioneine-loaded NIOs (steareth-2 as non-ionic surfactant, cholesterol, and cationic lipid) coated with HA.	❖ Antioxidative assays demonstrated that they can significantly scavenge ROS; ❖ Evaluation of expression level of tumor necrosis factor-α (TNF-α) and interleukin-6 (IL-6) in human dermal cells indicated the ↓↓ release of proinflammatory cytokines; ❖ Western blot detected ↓↓ MMP-3 expression levels in cells.	❖ Alternative for diseases with problematic drug penetration (cancers, skin conditions, and bacterial infections).	[105]
***Pulmonary DDS***	Hyaluronic acid-loaded NIOs (cholesterol, Span 80) were prepared using the ethanol injection method.	❖ DPPH assay proved ↑↑ antioxidant potential than free HA solution; ❖ In vitro hemolysis assay indicated low hemolysis values < 5%; ❖ In vivo pharmacokinetic studies indicated ↑↑ plasma concentration of HA compared to free HA solution administered via the aerosol route.	❖ Dual applicability in aerosol and i.v. route of administration.	[106]
***Cardiac DDS***	β-sitosterol-loaded NIOs (Tween 80, cholesterol, using the ether injection method, followed by ultrasonication) and coated with HA.	❖ ↓↓ lipid peroxidation; ❖ ↓↓ creatine kinase-MB, cardiac troponin-I (cTn-I), ↓↓ lactate dehydrogenase; ❖ ↑↑ ability to mitigate the adverse effects of cardiotoxicity.	❖ Treatment of cardiotoxicity and related cardiac conditions.	[15]

**Table 6:** Summary of NIO-ALG-DDS characteristics, preclinical evaluation, and applications.

Type of Niosomal ALG-DDS	Formulation Considerations	Preclinical Tests and Findings	Application	Ref.
***DDS for cancer therapy***	3D-printed gelatin-alginate scaffolds embedded with NIOs loaded with paclitaxel (cholesterol and Span 60) using thin-layer hydration method.	❖ The cytotoxicity analysis demonstrated 90% cytotoxicity against MCF-7 breast cancer cells; ❖ The flow cytometry results showed ↓↓ necrosis and ↑↑ apoptosis compared to free paclitaxel.	❖ Treatment of breast cancer and other cancers.	[110]
	pH-sensitive 3D-printed gelatin–alginate nanocomposites including NIOs (cholesterol and Span 60) loaded with doxorubicin using thin-layer hydration method.	❖ The scratch assay revealed a 60% ↓↓ in the covered surface area; ❖ ↑↑ 90% cell viability with MCF-10A nontumorigenic breast cells; ❖ ↑↑ 95% cell cytotoxicity against MCF-7 breast cancer cells.	❖ pH-responsive DDS for breast cancer treatment.	[111]
	NIO-based nanocarrier coated with alginate, designed for the co-delivery of doxorubicin (DOX) and cisplatin (CIS).	❖ MTT experiment indicated an IC_50_ ↓↓ than free drug; ❖ ↑↑ rate of apoptosis induction and induced cell cycle arrest in A2780 and MCF-7 cancer cells.	❖ Therapy of ovarian and breast malignancies.	[112]
	NIOs coated with calcium ALG encapsulating curcumin (Span 80, dicetyl phosphate, and cholesterol via thin-layer hydration method).	❖ ↑↑ biocompatibility with MCF-10A normal breast cells; ❖ ↑↑ cytotoxicity toward MD-MB-231 and SKBR3 breast cancer.	❖ Chemotherapy of breast cancer.	[113]
***Oral DDS***	Calcium alginate microspheres containing metformin-loaded NIOs (Span 60, dicetyl phosphate, and cholesterol via thin-layer evaporation method).	❖ In vivo studies on rats revealed ↑↑ of metformin hypoglycemic effect; ❖ Maintaining tight blood glucose levels over prolonged period of time after oral administration.	❖ Oral therapy of type 2 diabetes mellitus.	[114]

**Table 7:** Role of polysaccharides in niosomal formulations as DDSs.

Type of Niosomal DDS	Polysaccharide Used/Active Substance	Role of the Polysaccharide	Ref.
***Oral DDSs*** 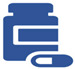	▪ ALG/metformin; ▪ CS/atorvastatin.	❖ Coating biopolymer; ❖ ↑↑ oral bioavailability, ↓↓ dosage and ↓↓ associated side effects, ↑↑ patient compliance.	[75,93,114]
	▪ O-palmitoyl PUL/tetanus toxoid antigen.	❖ Coating material; ❖ Viable oral vaccination delivery.	[75]
	▪ Fluorescein isothiocyanate-DXT (FITC-DXT)/ metformin hydrochloride+ +glipizide.	❖ Model of fluorescent stain for hydrophilic drugs used in encapsulation investigations; ❖ Observation of uniform distribution in inner core of the NIO of the hydrophilic drug (metformin hydrochloride).	[120]
***Cardiac DDSs*** 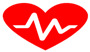	▪ HA/β-sitosterol;	❖ Coating biopolymer; ❖ ↑↑ specificity and ↑↑effectiveness of β-sitosterol in cardiac tissue.	[15]
***DDSs for cancer therapy*** 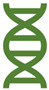	▪ CS/epirubicin/5-fluorouracil/finasteride.	❖ Coating biopolymer of the nanoparticles of epirubicin; ❖ Targeted treatment of breast cancer cells/prostate cancer prophylaxis.	[83,87]
	▪ HA/alpha-terpineol/epirubicin/curcumin/doxorubicin.	❖ Coating biopolymer; ❖ Selectively binding to cell surface adhesion receptor CD44 strongly expressed in many types of cancer; ❖ ↑↑ the intracellular transport of anticancer drugs to numerous malignant cells.	[3,100]
	▪ FITC-DXT/doxorubicin+ curcumin.	❖ Model of fluorescent stain for hydrophilic drugs used in encapsulation investigations; ❖ Observing the uniformity distribution in inner aqueous core of the NIO of the hydrophilic drug (doxorubicin).	[119]
	▪ Calcium ALG/curcumin.	❖ Coating biopolymer; ❖ ↑↑ efficacy of chemotherapy.	[113]
***Pulmonary DDSs*** 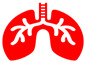	▪ Aerosolized HA-loaded NIOs.	❖ Coating biopolymer; ❖ ↑↑ localization of HA in the lungs; ❖ The trapping of HA inhibits its breakdown by the enzymes found in surrounding cells.	[106]
***Ocular DDSs*** 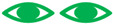	▪ CS/itraconazol.	❖ Coating biomaterial; ❖ ↑↑ the ocular absorption; ↑↑ drug’s ocular permeability	[96]
	▪ HA/tacrolimus.	❖ Coating biomaterial; ❖ ↑↑ ophthalmic bioavailability↑↑ corneal permeability; ❖ ↑↑ mucoadhesion on the ocular mucosa.	[13]
***Gene DDSs*** 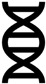	▪ CS N-arginine/plasmid DNA.	❖ Coating biomaterial with different degree of substitution; ❖ ↑↑ transfection efficiency.	[90]
***Nose-to-brain DDSs*** 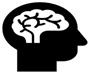	▪ CS/clonazepam/ /lacosamide.	❖ Coating biomaterial for prolonged release profile; ❖ ↑↑ bioavailability, ↑↑ nasal diffusion, ↑↑ brain distribution following intranasal delivery.	[91,92]
***Transdermal DDSs*** 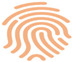	▪ CS/moxifloxacin/ /cefazolin.	❖ Gel former for prolonged and constant drug release; ❖ ↑↑ bio adhesive properties, ↑↑ local retention, ↑↑ efficacy	[94,95]
	▪ HA/ergothioneine.	❖ Gel former, concentrated the drug in the targeted cellular locations; ❖ ↑↑ capacity to traverse transdermal biological barrier.	[105]

## Data Availability

The data may be requested from the authors.
